# Tropical *Bacillus* as Hidden Producers of Thermostable and Structurally Complex Exopolysaccharides

**DOI:** 10.1021/acsomega.6c00394

**Published:** 2026-04-30

**Authors:** Jessica Silva de Almeida, Matheus de Oliveira Barros, Lorena Mara Alexandre e Silva, Arcelina Pacheco Cunha, Christiana de Fátima Bruce da Silva, Morsyleide de Freitas Rosa, Ana Iraidy Santa Brígida

**Affiliations:** † Federal University of Ceará (UFC), Department of Chemical Engineering, Fortaleza 60881-905, Ceara, Brazil; Embrapa Tropical Agroindustry, Rua Dra Sara Mesquita 2270, Fortaleza 60511-075, Ceara, Brazil; § State University of Ceara (UECE), Center for Equipment and Laboratories and Multi-User Advanced Microscopy Center, Fortaleza 60714-903, Ceara, Brazil

## Abstract

Exopolysaccharides (EPS) produced by microorganisms are increasingly valued as sustainable biopolymers, yet the properties of EPS derived from bacteria adapted to tropical agroecosystems remain underexplored. Tropical *Bacillus* strains, specifically those adapted to the harsh, high-temperature environments, represent a critical and underexplored reservoir of robust biopolymers. This study investigated four *Bacillus* sp. isolates obtained from banana plantation soils in Northeastern Brazil, demonstrating their inherent potential to synthesize structurally complex EPS. The produced polymers were purified and characterized using spectroscopic, chromatographic, and thermal analyses to determine their chemical composition and physicochemical behavior. The EPS produced by the isolates were identified as heteropolysaccharides with variable monosaccharide profiles, indicating strain-dependent biosynthetic pathways. Thermal analysis revealed notable thermal stability (onset of degradation near 240 °C), with degradation patterns suggesting a high degree of structural organization compared with many previously reported microbial EPS. These differences highlight the influence of ecological adaptation on polymer architecture and functionality. Overall, the findings demonstrate that tropical Bacillus strains represent promising and underutilized sources of EPS with properties suitable for applications requiring resistance to thermal processing. This work expands the understanding of environmentally adapted microbial biopolymers and supports their potential use in sustainable industrial and biotechnological materials.

## Graphical abstract

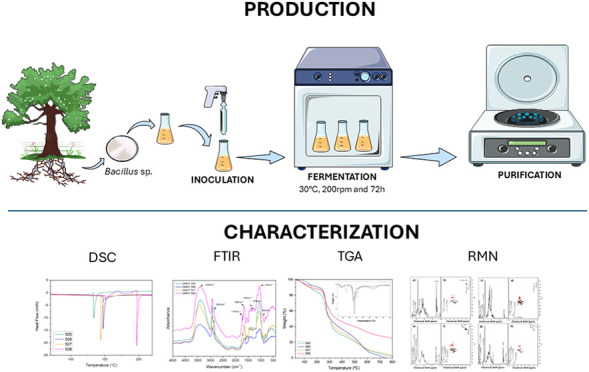

## 1 Introduction

Exopolysaccharides (EPS) are high-molecular-weight biopolymers, ionic or nonionic and often branched, secreted by bacteria, algae, yeasts, and fungi. EPS synthesis depends on a set of enzymes that may vary among species and strains. These differences determine the formation of precursor nucleotide sugars and the activity of glycosyltransferases responsible for the assembly and polymerization of repeating units, resulting in polymers with different compositions and structures.^1^ EPS can be classified into two groups: homopolysaccharides, composed of a single type of monosaccharide, and heteropolysaccharides, which contain multiple monosaccharides and may vary in branching and chain length.^2^

EPS properties are intrinsically linked to their monosaccharide composition and structure.^3^ An example is the study by Guan et al.,^4^ in which an EPS was produced from *Lacticaseibacillus rhamnosus* mainly composed of arabinose, glucose, and fructose, that exhibited significant antioxidant activity, demonstrated by total antioxidant capacity assays and concentration-dependent free radical scavenging. The authors attributed this effect to the abundance of hydroxyl groups capable of stabilizing free radicals. Such structural differences directly influence physicochemical properties and potential applications.

Microbial EPS have gained relevance in food, chemical, cosmetic, and pharmaceutical industries due to their emulsifying, stabilizing, flocculating, film-forming, and encapsulating capacities.^5–8^ Several studies highlight microbial EPS for their thermal stability, rheological functionality, and bioactivities, positioning them as promising natural additives for functional foods and technological applications. Several studies also highlight the thermal stability of these biopolymers as an important characteristic for technological applications, particularly when subjected to processes involving heat, such as sterilization, drying, extrusion, or film formation. In different investigations, microbial EPS have exhibited initial degradation temperatures in the range of 180 to 200 °C, and such stability is considered a relevant criterion for their application in industrial systems.^9,10^

Thermal stability is particularly important because it determines the material’s ability to maintain its structural and functional integrity under severe processing conditions. Polymers that undergo premature degradation may lose viscosity, emulsifying capacity, or mechanical properties, thereby compromising their final performance. Therefore, EPS with greater thermal resistance expand the range of potential industrial applications, enhancing their scalability and technological feasibility.^10^

In addition, bioactivities such as antioxidants, immunomodulatory, anticancer, antifungal, hypoglycemic, and antimicrobial effects have further expanded interest in these biopolymers.^11,12^ Well-known examples: xanthan, gellan, carrageenan, pullulan, levan, and Curdlan; are already widely used industrially.^13^ Among natural polysaccharides, sodium alginate also stands out, being widely explored due to its biocompatibility, film-forming ability, and structural versatility, with numerous applications in biomedical systems, functional hydrogels, and sensor devices. Recent studies demonstrate that its structural properties directly influence its mechanical, thermal, and functional performance in advanced materials.^14,15^

Although various microorganisms produce EPS, *Bacillus* species have gained prominence because they are easy to ferment and broadly applicable. Bacillus strains stand out for contributing to soil water retention, promoting plant growth, and mitigating drought stress.^16^ Many *Bacillus* species are thermophilic, and their EPS often exhibit high thermal stability.^17^ Their ability to form spores provides resistance to adverse environmental conditions, facilitating storage, optimizing production, and enabling their use in microbial inoculants.^18^

Several strains have been reported in the literature as producers of EPS with properties of industrial interest. Among them, *Bacillus haynesii* CamB stands out as a producer of a heteropolysaccharide EPS composed of mannose, glucose, and galactose, notable for its high water-holding and emulsifying capacities.^17^ EPS from B. cereus have demonstrated the ability to enhance soil moisture retention^16^ and even showed potential for treating staphylococcal infections.^19^

Despite these advances, the biotechnological potential of *Bacillus* strains adapted to tropical soils, particularly those exposed to heat, drought, and low water availability, remains little explored. Tropical environments exert selective pressures that often favor microorganisms capable of producing structurally robust and thermally stable EPS as survival strategies. Still, few studies have investigated native *Bacillus* from such ecosystems as sources of novel biopolymers.^20^

This knowledge gap is especially relevant in Northeastern Brazil, a region characterized by high temperatures and fluctuating soil moisture levels, conditions that may naturally select bacteria with enhanced EPS biosynthesis capabilities.

In this context, the present study aimed to investigate whether four native *Bacillus* sp. isolates obtained from banana plantation soils in the Northeastern region of Brazil are capable of producing exopolysaccharides (EPS). To address this objective, the produced polymers were subjected to chemical, structural, and thermal characterization, and the results were compared with data available in the literature.

The findings confirmed that the evaluated strains synthesize heteropolysaccharidic EPS with distinctive structural complexity and high thermal stability, exhibiting degradation onset temperatures higher than those commonly reported for microbial EPS. These results demonstrate not only the biosynthetic capacity of these tropical strains but also indicate that the produced polymers possess differentiated thermally stable properties, positioning these isolates as a promising source of robust and technologically relevant biopolymers.

## 2 Materials and Methods

### 2.1 Materials

The culture media used in this study included NYD (Nutrient Yeast Dextrose) medium and NYDA (Nutrient Yeast Dextrose Agar), commonly employed for the growth and maintenance of the strains. The medium described by Rodríguez and Callieri was used, a formulation widely applied for EPS production, composed of yeast extract as a nitrogen source, sucrose as the primary carbon source, and mineral salts to support microbial growth and polysaccharide biosynthesis.^21^ The following analytical-grade reagents and components were employed in media preparation and subsequent analytical procedures: dextrose, yeast extract, meat extract, meat peptone, ammonium sulfate, magnesium sulfate heptahydrate, agar, sucrose, and dipotassium phosphate. Ethanol (analytical grade) and trichloroacetic acid (TCA) were used for EPS precipitation and purification steps.

### 2.2 Microorganisms

The bacterial strains used in this study (CMIAT 505, 506, 507 and 508) were obtained from the Microorganism Collection of Embrapa Tropical Agroindustry (CNPAT), Fortaleza, CE, Brazil. These strains were originally isolated from banana plantation soils in Northeastern Brazil and are preserved as part of the institutional microbial collection.

The *bacillus* sp. strains were stored in nutrient-yeast-dextrose (NYD) broth supplemented with 15% glycerol and stored at −80 °C.

Although the original isolation procedures were not performed within the scope of this study, the strains are curated and maintained under controlled conditions in the collection.

### 2.3 Maintenence

The strains were cultivated in NYD/NYDA medium for activation and maintenance, with the following composition: dextrose (10 g/L; 99.5% purity, Vetec, Brazil); yeast extract (5 g/L; reagent grade, Kasvi, Brazil); meat extract (3 g/L; reagent grade, HiMedia, India); meat peptone (5 g/L; reagent grade, Sigma-Aldrich, USA); ammonium sulfate ((NH_4_)_2_SO_4_, 3 g/L; 99% purity, Vetec, Brazil); magnesium sulfate heptahydrate (MgSO_4_·7H_2_O, 0.5 g/L; 99.5% purity, CRQ-LTDA, Brazil); and agar (15 g/L; reagent grade, Dinâmica, Brazil) for solid medium.^22^

### 2.4 Fermentation

Fermentation was carried out in the medium described by Rodríguez & Callieri (1986),^23^ composed of yeast extract (5 g/L; reagent grade, Kasvi, Brazil), sucrose (50 g/L; 99.5% purity, Dinâmica, Brazil), ammonium sulfate (1 g/L; 99.5% purity, Vetec, Brazil), magnesium sulfate heptahydrate (1 g/L; 99.5 purity, CRQ-LTDA, Brazil), and monobasic potassium phosphate (1 g/L; 99% purity, Vetec, Brazil). The individual components of the medium were weighed separately, dissolved in distilled water to the previously calculated final volume, and subsequently sterilized.^24^ The experiment was conducted individually for each strain, with 25 samples prepared per strain. In the initial stage, the inoculum was prepared in a shaker incubator under aerobic conditions, using 250 mL Erlenmeyer flasks containing 50 mL of culture medium, for 24 h at 30 °C and 150 rpm. Subsequently, the sterile medium was inoculated at 10% (v/v), and fermentation was conducted under the same conditions for 72 h at 30 °C and 150 rpm.

### 2.5 EPS Purification

After fermentation, 10% (w/v) trichloroacetic acid (TCA), relative to the culture volume, was added to each flask, and the biomass was separated from the supernatant by centrifugation for 15 min at 3600 rpm and room temperature. Analytical grade ethanol was then added to the supernatant at a 2:1 ratio, and the mixture was left to sit for 24 h to allow EPS precipitation. Residual ethanol was removed through successive centrifugation steps, after which 30 mL of water and 60 mL of ethanol were added to the precipitated EPS and the mixture was again left to precipitate for 24 h. The material was then centrifuged once more and lyophilized for subsequent characterization.

### 2.6 Characterization

#### 2.6.1 Determination of EPS Yield and Productivity

Yield was calculated as the ratio between the dry mass of EPS obtained and the volume of culture medium used in the fermentation, expressed in g/L. From this value, productivity was determined as the production divided by the total duration of the fermentation, in days, with results expressed in g/L·day.

#### 2.6.2 Sugar Content and Consumption

Total sugar content was determined according to the phenol–sulfuric acid method described by Dubois et al. (1956), using sucrose as the equivalent standard. Absorbance was measured in a FEMTO Cirrus 60ST spectrophotometer (São Paulo, Brazil) at 490 nm. All analyses were performed at Embrapa Agroindústria Tropical, Brazil.

Sugar consumption (%) was determined by using the following eq 1

1 $$\mathrm{sugar}\;\mathrm{consumption}\;\left(\%\right)=100-\left(\frac{100\times S_{f}}{S_{i}}\right)$$

where *S*_f_ is the concentration of sugar after fermentation and *S*_i_ is the initial concentration of sugar in the culture medium (50 g/L) for the Rodriguez and Callieri medium.

#### 2.6.3 Fourier Transform Infrared Spectroscopy (FTIR)

To identify the main functional groups, present in the samples and relate them to typical EPS groups, FTIR analysis was performed using a PerkinElmer Spectrum Two instrument (Connecticut, USA). Dried EPS (1 mg) was mixed with KBr powder (99 mg), ground, and pressed into a 7 mm pellet. Spectra were collected from 450 to 4000 nm with a resolution of 4 cm^–1^.^25^ The analyses were conducted in triplicate at Embrapa Agroindústria Tropical, Brazil.

#### 2.6.4 Thermogravimetric Analysis (TGA)

Thermal stability was evaluated by thermogravimetric analysis (TGA) using a PERKIN ELMER STA 6000 (Connecticut, USA) instrument with approximately 10 mg of sample. The analyses were conducted at a heating rate of 10 °C/min, over a temperature range of 50 to 800 °C, under a nitrogen atmosphere with a gas flow rate of 40 mL/min. Each sample was analyzed once, and the presented curves are representative of the observed thermal behavior. All analyses were performed at the Laboratory of Products and Process Technology (LPT), Federal University of Ceará (UFC), Brazil.

#### 2.6.5 Differential Scanning Calorimetry (DSC)

DSC analysis was performed using a TA Instruments Q20 DSC (New Castle, USA) at Embrapa Agroindústria Tropical, Brazil, with samples placed in hermetically sealed pans. Approximately 2 mg of sample was used, and measurements were conducted from 25 to 300 °C at a heating rate of 10 °C/min under a nitrogen atmosphere.

#### 2.6.6 Gas Chromatography–Mass Spectrometry (GC-MS)

Samples were derivatized according to the procedure described by Menezes et al.^21^ Analyses were performed at Embrapa Agroindústria Tropical, Brazil, using an Agilent 5977A GC-MS (California, USA) system equipped with an HP-5MS fused-silica capillary column (30 m × 0.25 mm i.d., 0.25 μm film thickness) and a quadrupole mass analyzer operating in electron ionization (EI) mode at 70 eV, with a mass scan range of 50–600 *m*/*z* and an acquisition rate of 2.7 scans·s^–1^. Injections were performed in split mode (1:20) with an injection volume of 1 μL, using helium as the carrier gas at 1 mL·min^–1^. Injector and interface temperatures were set to 250 and 280 °C, respectively. The oven program started at 190 °C (held for 4 min), followed by a ramp to 230 °C at 4 °C·min^–1^, and a final temperature of 250 °C held for 8 min. Peak identification was based on the fragmentation patterns of injected sugar standards.

#### 2.6.7 Nuclear Magnetic Resonance (NMR) Spectroscopy

NMR spectra were acquired at Embrapa Agroindústria Tropical, Brazil, on an Agilent DD2 600 MHz spectrometer (^1^H frequency) (California, USA), equipped with a 5 mm One Probe (H–F/^15^N–^31^P) for inverse detection and a *z*-axis field-gradient system. Approximately 10 mg of sample was dissolved in 600 μL of D_2_O (99.9%) containing 0.1% sodium 3-(trimethylsilyl)­propionate-*d*_4_ (TMSP-*d*_4_) and transferred to a 5 mm NMR tube. One-dimensional ^1^H spectra were recorded with a 2 s relaxation delay, 3.3 s acquisition time, receiver gain of 34, 32 transients, a spectral width of 16 ppm, and 32k real points at 60 °C. Structural elucidation of the components was supported by two-dimensional NMR experiments, including homonuclear ^1^H–^1^H correlation (COSY), heteronuclear single-quantum ^1^H–^13^C correlation (HSQC), and heteronuclear multiple-bond ^1^H–^13^C correlation (HMBC), interpreted with reference to the relevant literature.^4–6,26–28^

## 3 Results and Discussion

### 3.1 EPS Yield, Productivity, and Sugar Consumption

EPS production varied among the evaluated strains (Table 1). Strain CMIAT 505 exhibited the highest EPS yield (0.133 g/L), whereas strain CMIAT 508 showed the lowest value (0.062 g/L). Strains CMIAT 506 and 507 presented intermediate yields of 0.107 and 0.095 g/L, respectively.

**Table 1:** Yield, Productivity and Sugar Consumption for EPS Samples Obtained from the Fermentation of Strains CMIAT 505, 506, 507, and 508

samples	yield (g/L)	productivity (g/L·day)	sugar consumption (%)
505	0.133 ± 0.008	0.044 ± 0.003	89.68 ± 1.29
506	0.107 ± 0.014	0.036 ± 0.005	90.26 ± 0.46
507	0.095 ± 0.007	0.032 ± 0.002	75.90 ± 6.44
508	0.062 ± 0.010	0.021 ± 0.003	69.13 ± 7.75

A similar trend was observed for productivity. Strain CMIAT 505 again showed the highest rate (0.044 g/L·day), followed by strains CMIAT 506 (0.036 g/L·day) and CMIAT 507 (0.032 g/L·day). Strain CMIAT 508 displayed the lowest productivity (0.021 g/L·day).

The comparison highlights a significant aspect of the current work, even though these values are lower than EPS yields reported in studies using optimized conditions or enriched media (3–8 g/L),^19,29^ the comparison highlights an important aspect of the present work. Unlike standardized laboratory strains, the *Bacillus* isolates examined here were obtained from tropical soils and cultivated under baseline fermentation conditions not tailored to their physiology. Even so, the strains consumed substantial amounts of the available carbon source, reducing sucrose from 50 g/L to as low as 4.5 g/L, indicating active metabolism and demonstrating that carbon flux is occurring, albeit likely diverted toward cellular maintenance rather than polysaccharide biosynthesis.

This finding presents a critical opportunity: the low EPS yield is indicative of suboptimal cultivation parameters rather than an intrinsic limitation of the strains. Native microorganisms often require specific nutrient balances distinct from traditional industrial strains. Therefore, given the substantial sugar consumption, it is highly probable that EPS production can be significantly amplified through process engineering strategies, such as altering carbon-to-nitrogen ratios, modulating osmotic conditions, or exploring substrates that better mimic their tropical ecological niche. The isolates’ capacity to produce structurally complex and remarkably thermostable polymers even under nonideal baseline conditions positions them as promising and robust candidates for intensive bioprocess development.

Beyond quantitative enhancement, EPS optimization strategies also influence molecular architecture and functional performance. Previous studies have demonstrated that substrate composition, nutrient balance, and environmental parameters can modulate molecular weight distribution, functional group composition, and branching patterns of microbial polysaccharides, directly affecting their physicochemical behavior and application potential. Thus, optimization should not be viewed solely as a productivity-driven objective, but as a tool to rationally direct carbon flux toward the synthesis of polymers with tailored structural and thermal properties. In this context, the high carbon consumption observed in the present study suggests that metabolic regulation, rather than substrate limitation, governs EPS yield under baseline conditions, reinforcing the relevance of future process design approaches.^30,31^

### 3.2 Fourier Transform Infrared Spectroscopy (FTIR)

The infrared absorption spectrum of a compound is often described as its molecular fingerprint and is typically the first tool used for the structural characterization of biopolymers.^32^

The FTIR spectra obtained for all four EPS samples (Figure 1) confirmed their polysaccharide nature and verified EPS production by all strains. Polysaccharides commonly display a broad absorption band above 3390 cm^–1^, corresponding to O–H stretching vibrations from hydroxyl groups in their constituent monosaccharides. Additionally, the band near 2800 cm^–1^ is attributed to C–H stretching, indicating the presence of methyl and methylene groups, typically found in hexoses such as glucose and galactose.^33^

**Figure 1:**
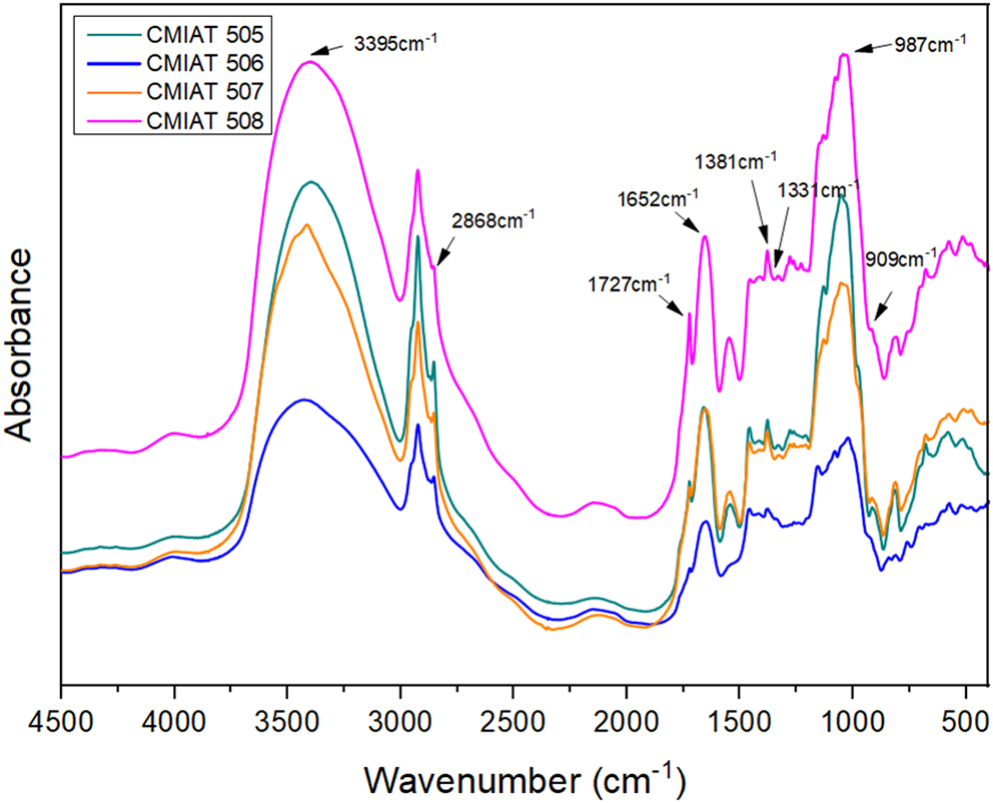
FTIR spectra of EPS samples obtained from the fermentation of strains CMIAT 505, 506, 507, and 508.

The band around 1652 cm^–1^ region may be associated with the polymer’s interaction with water through C–H bonding. In other cases, it may be related to C–H stretching vibrations and the presence of carboxylic groups within the saccharide ring. However, for microbial EPS, the assignment to the deformation of adsorbed water is the most widely accepted, although contributions from organic functional groups may coexist depending on the composition of the sample.^34–36^

Bands near 1700 cm^–1^ (1727 cm^–1^ in Figure 1) correspond to R–C=O stretching vibrations associated with organic acids commonly found in microbial EPS, such as pyruvic and uronic acids.^37^ The band at approximately 1380 cm^–1^ is associated with C–C stretching, while the band near 1338 cm^–1^ corresponds to C–O bending vibrations. The absorption at around 987 cm^–1^ indicates C–O–C ether linkages, characteristic of carbohydrate structures.^38^

In the fingerprint region (<1500 cm^–1^), the presence of bands around 909 cm^–1^ is indicative of glycosidic linkages, commonly reported in certain glucans.^39^ Overall, the spectra of the analyzed samples exhibit the typical features of bacterial exopolysaccharides, and the absence of nitrogen-containing bands indicates that no cellular residues remained in the purified material.

### 3.3 Thermogravimetric Analysis (TGA)

The thermogravimetric analysis (TGA) curves (Figure 2) describe the thermal decomposition profile of the natural polymers produced, recording mass loss as temperature increases at a constant rate. Because thermal behavior is a key parameter for determining the industrial applicability of a material, TGA is a valuable tool for assessing its thermal properties.

**Figure 2:**
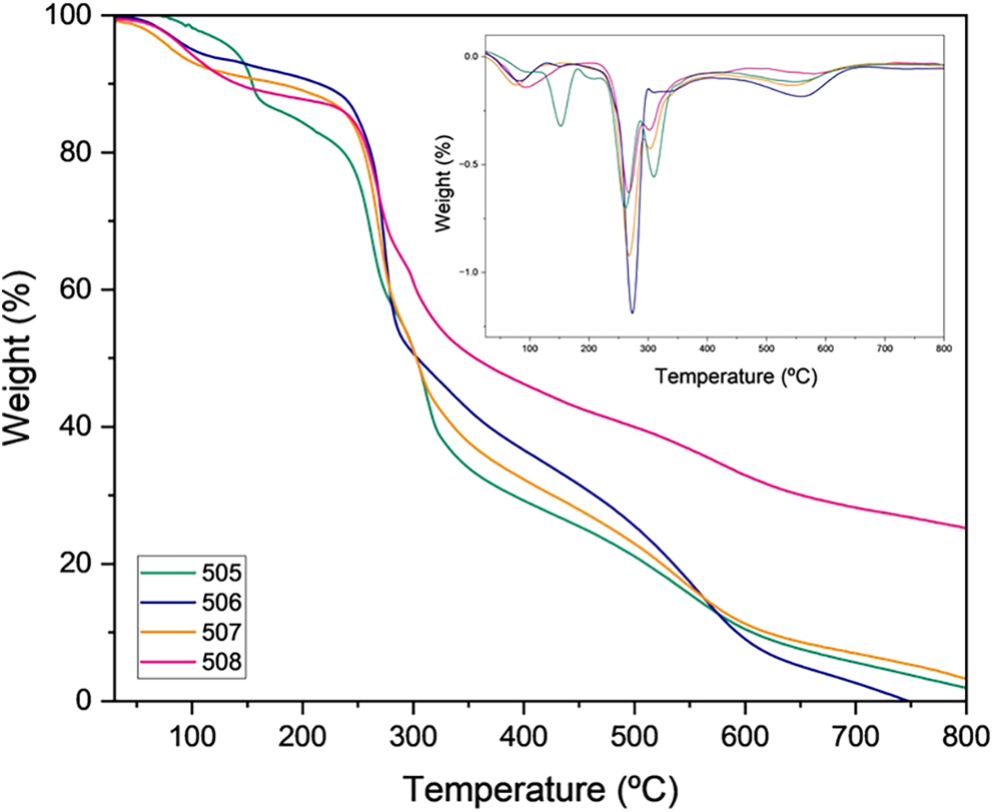
Thermograms of EPS samples obtained from the fermentation of strains CMIAT 505, 506, 507, and 508.

All samples displayed the expected initial mass loss of approximately 8% near 100 °C, attributable to the removal of residual moisture. However, the thermal stability profile demonstrated a highly significant finding: the onset of thermal degradation for all strains occurred between 237 and 244 °C (Table 2), with the average onset temperature consistently near 240 °C. These values fall within the range reported for microbial exopolysaccharides, for which considerable variations in thermal stability have been described. Studies report lower initial degradation temperatures in the range of 180 to 200 °C.^9,10^ While other works describe EPS with higher thermal stability, such as EPS produced by *Pediococcus pentosaceus* E8, with stability up to approximately 257 °C,^34^ and EPS from *Leuconostoc pseudomesenteroides* JF17, with degradation temperatures around 320 °C.

**Table 2:** Thermogravimetric Data for EPS Samples Obtained from the Fermentation of Strains CMIAT 505, 506, 507, and 508

sample	*T*_onset_ (°C)	1st event	2nd event	ash content (%)
505	239	260	310	1.8
506	243	272	308	2.9
507	237	268	301	3.3
508	244	266	301	25.5

Therefore, the thermal behavior observed in this study, with values around 240 °C, can be positioned within an intermediate range, indicating relevant thermal stability consistent with EPS reported in the literature.

This differentiated thermal behavior may be associated with the structural features identified by GC–MS and NMR analyses. GC–MS confirmed the heteropolymeric nature of the EPS, revealing four different types of monosaccharides, which increases structural complexity. Such heterogeneity may result in different types of glycosidic linkages and variations in polymer chain organization, directly influencing the energy required for thermal bond cleavage. In this context, the exceptionally enhanced thermal stability observed in these materials may reflect the intrinsic robustness of polymers synthesized by native *Bacillus* strains adapted to tropical environmental stressors. This structural resilience strongly supports the potential application of these biopolymers in high-temperature processing environments where maintaining material integrity is essential.

Residual mass (Ash content) results showed low levels for strains 505, 506, and 507 (1.8% to 3.3%). In contrast, strain 508 exhibited an exceptional residual mass of 25.5%. Similar high residue levels have been described by Wei et al.^7^ (28%) and Jiang et al.^40^ (33.9%). Such elevated residual content is typically associated with thermally resistant components, including mineral salts and ash.^41^

The main degradation event, characterized by substantial mass loss, occurred in multiple stages. This multistep pattern is consistent with the heteropolymeric nature of the EPS, confirmed by GC–MS analysis, and reflects the greater structural complexity of heteropolysaccharides compared with homopolysaccharides.

### 3.4 Differential Scanning Calorimetry (DSC)

The DSC analysis revealed endothermic peaks ranging from 134 °C (strain 505) to 197 °C (strain 508), indicating differences in chain mobility and structural organization among the exopolysaccharides (EPS) (Figure 3). The EPS produced by strain 505 exhibited the lowest endothermic peak (134 °C), suggesting a more flexible and less ordered structure. Strains 506 and 507 showed similar thermal behavior, with peaks at 147.6 and 144.0 °C, respectively, indicating an intermediate level of structural organization. In contrast, the EPS from strain 508 demonstrated the highest rigidity and thermal stability, presenting two distinct high-temperature endothermic events at 182.9 °C (Δ*H* = 71.9 J/g) and 197.0 °C (Δ*H* = 120.3 J/g).^17^

The thermal events observed in samples 505, 506, and 507 around 140 °C (Table 3) are likely associated with melting processes that precede the onset of thermal degradation. This distinct thermal profile for strain 508 correlates strongly with the TGA findings, which revealed its exceptionally high ash content (25.5%). The initial minor event at 182.9 °C is potentially related to these residual salts and inorganic components. A secondary, less intense endothermic event was detected around ∼150 °C in all samples, including EPS 508. This transition is interpreted as a minor structural relaxation or dehydration-associated rearrangement, commonly reported for polysaccharides containing bound water or less-ordered regions, and does not represent the principal thermal transition of the material. We suggest that these high levels of inorganic residues may function as protective agents, physically interfering with the initial depolymerization step and subsequently elevating the major thermal transition peak to the higher temperature of 197 °C higher temperature (197 °C).^42^

**Table 3:** DSC Data for EPS Samples Obtained from the Fermentation of Strains CMIAT 505, 506, 507, and 508

sample	*T*_max_ (°C)	Δ*H*_melt_ (J/g)
505	133.9	116.0
506	147.6	152.1
507	144.0	148.0
508	182.9/197.0	71.9/120.3

TGA analysis shows that all samples began thermal degradation only above 238 °C, supporting the DSC findings. Together, these results indicate that the produced EPS exhibit good thermal stability, a relevant property for applications requiring heat resistance.^43^

### 3.5 Gas-Chromatography and Nuclear Magnetic Resonance

The four exopolysaccharide samples analyzed were identified as heteropolysaccharides, composed predominantly of d-mannose, d-glucose, d-galactose, and d-fructose. The main backbone consisted of mannose and glucose units linked through α- and β-glycosidic bonds of the (1→2), (1→4), and (1→6) types.^7,27,44^

GC–MS analysis (Table 4) revealed variations in monosaccharide composition, suggesting that the heteropolysaccharide nature of the samples may be associated with different biosynthetic pathways or culture conditions.^26^

**Figure 3:**
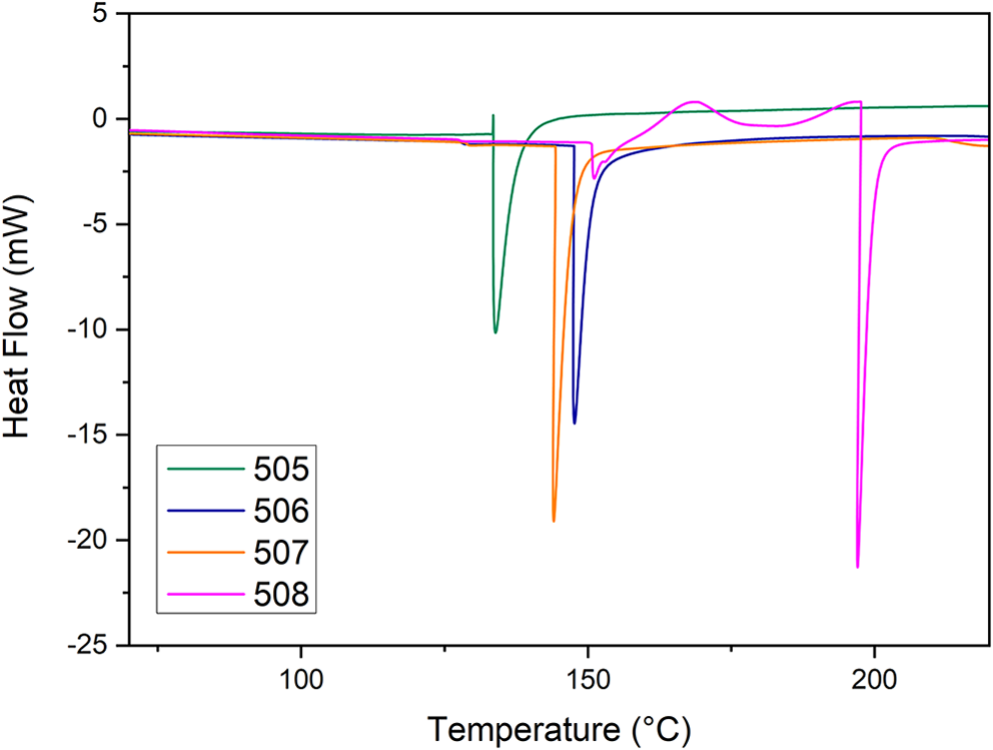
Differential scanning calorimetry (DSC) heat-flow curves of EPS obtained from the fermentation of strains CMIAT 505, 506, 507, and 508.

**Table 4:** Monomers Identified by Gas Chromatography–Mass Spectrometry (GC–MS) in the EPS 505, EPS 506, EPS 507, and EPS 508 Samples

	relative percentage (%)				monosaccharides ratio			
EPS	505	506	507	508	505	506	507	508
manose	37.6	30.1	29.5	41.9	5	4	2	4
fructose	15.6	22.2	12.8	11	2	3	1	1
glucose	36	31.8	38.5	17.1	5	4	3	2
galactose	7.8	7.3	14	13.5	1	1	1	1

Analysis of the NMR spectra (Figure 4) confirmed the presence of both α- and β-anomeric configurations (δ 4.8–5.6 ppm) and carbon signals characteristic of pyranoside units (98–106 ppm, HSQC). All samples exhibited detectable acetyl and methyl groups, indicating partial substitutions that may influence solubility and certain functional properties. The presence of O–CH_3_ groups (δ ∼ 2.0–2.6 ppm; ^13^C ∼ 170–175 ppm) was further supported by HMBC correlations, confirming the acetylation of sugar residues.^6^

**Figure 4:**
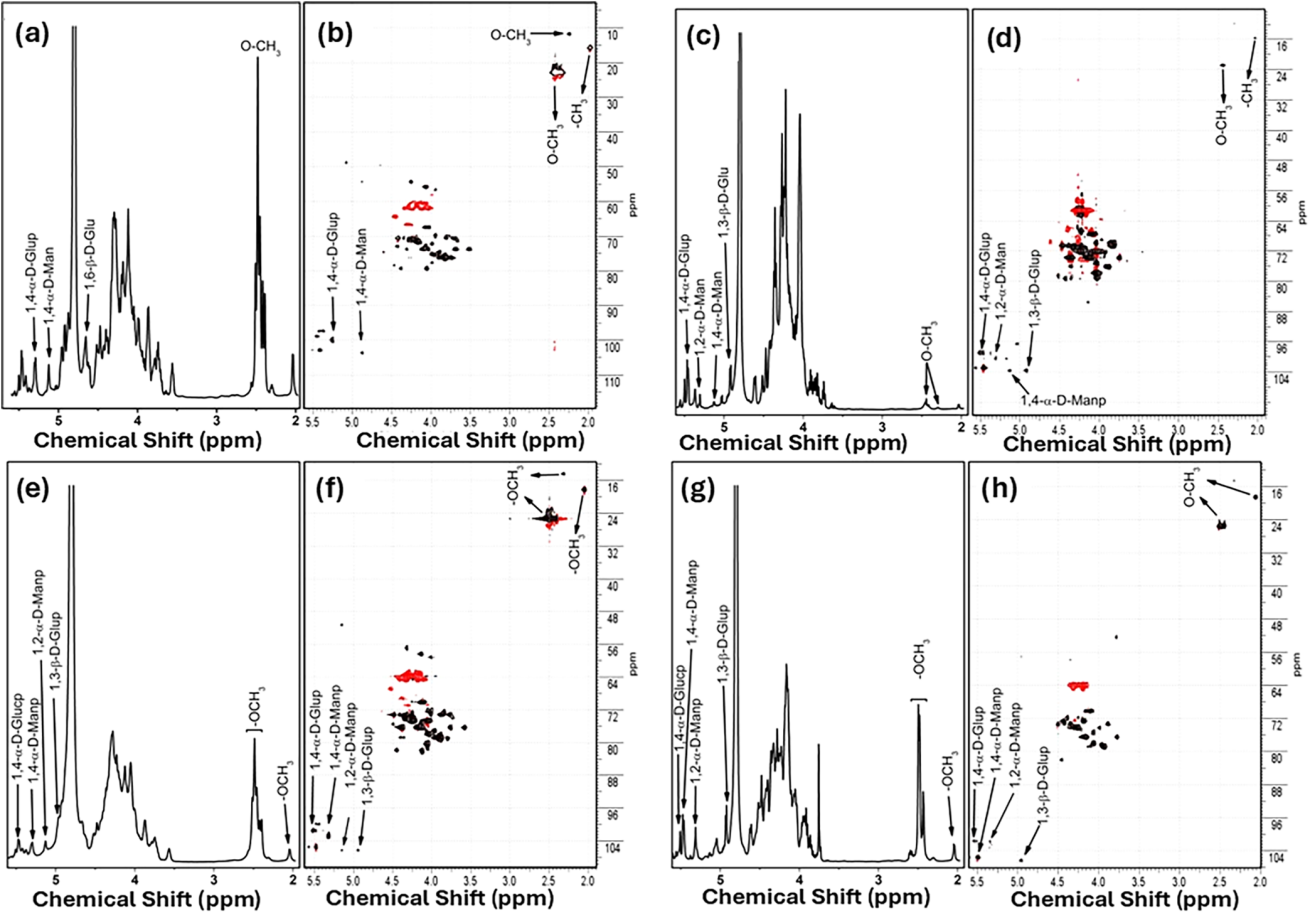
NMR spectra of the EPS samples. EPS 505: (a) ^1^H NMR and (b) ^1^H–^13^C HSQC; EPS 506: (c) ^1^H NMR and (d) ^1^H–^13^C HSQC; EPS 507: (e) ^1^H NMR and (f) ^1^H–^13^C HSQC; EPS 508: (g) ^1^H NMR and (h) ^1^H–^13^C HSQC.

The glucose/manose ratios were similar in EPS 505 and EPS 506, and the moderate levels of galactose and fructose suggest the presence of glucogalactan-type branches linked through (1→3), (1→4), and (1→2,6) glycosidic bonds.^27,45^ EPS 507 exhibited a higher proportion of glucose (38.5%) and galactose (14%), whereas EPS 508 was enriched in mannose (41.9%) and showed a lower glucose content (17.1%).

Although NMR provided detailed information regarding substitution patterns and glycosidic connectivity, GC–MS played a key complementary role by enabling quantification of the monosaccharide composition and confirming the heteropolysaccharide nature of the samples. Together, these analyses demonstrate that all EPS samples are complex, branched heteropolysaccharides composed primarily of glucose and mannose, partially substituted with galactose, fructose, and acetyl groups, which confer distinct structural and functional characteristics.^4–6^

## 4 Conclusion

This foundational study successfully demonstrated that native *Bacillus* strains, specifically those isolated from tropical banana plantation soils, can produce structurally complex and highly thermostable exopolysaccharides. Structural elucidation confirmed that all four strains synthesize branched heteropolysaccharides rich in mannose and glucose, incorporating valuable features such as acetylated residues that contribute to their functional and technological relevance. The primary and most significant finding is the exceptional thermal behavior of these materials. Thermal degradation, with an onset consistently near 240 °C, is substantially higher than the values commonly reported for standard microbial EPS. This superior heat resistance is a direct reflection of the natural robustness conferred by the adaptation of these microorganisms to harsh tropical environmental conditions. Although EPS yields were initially modest, this deficit is clearly linked to the use of nonoptimized, standard fermentation media, rather than a lack of intrinsic biosynthetic potential. The active sugar consumption observed indicates that metabolic flux is occurring and can be effectively redirected toward high polysaccharide production through strategic process engineering. In summary, this work establishes a critical foundational map for future bioprocess development. It clarifies the remarkable chemical and thermal potential of *Bacillus* strains from Northeastern Brazilian soils and defines the subsequent research path required, specifically, the optimization of cultivation conditions, to unlock the high productivity needed to transform these naturally robust EPS into competitive, heat-resilient biobased materials.
